# Continuous efforts in the clinical and paraclinical management of the human brain pathology

**Published:** 2014

The 10th International Congress of the Society for the Study of Neuroprotection and Neuroplasticity (SSNN) took place in the elegant and well-equipped halls of Hilton Hotel, in Athens, between October 30 and November 2, 2014 and presented the following major themes: secondary prevention of stroke and the progresses that were lately registered in neurology.

Before the start of the event, the President of SSNN and the Neurology Society in Romania, Prof. Dafin Muresanu, MD, declared *“The SSNN Congress had come to the 10th edition and, just like each time, enjoyed the participation of a large number of medical personalities from Europe, USA and Asia and last, but not least, from Romania. The scientific program was comprehensive; the main themes discussed being about the latest discoveries in the vascular cerebral diseases, craniocerebral trauma and neurodegenerative diseases. The importance of the congress was remarkable because it reunited people with a vast professional experience who managed to integrate the information offered from different perspectives as a whole. These scientists tried to decode the molecular mechanisms of the neurological diseases, their discoveries representing the basis of the precocious diagnosis and the efficient treatment. Moreover, it is important to mention the contribution of the Romanian neurology in this event, proving this way, that we have valuable people, whose value is internationally recognized”*.

**Fig. 1:**
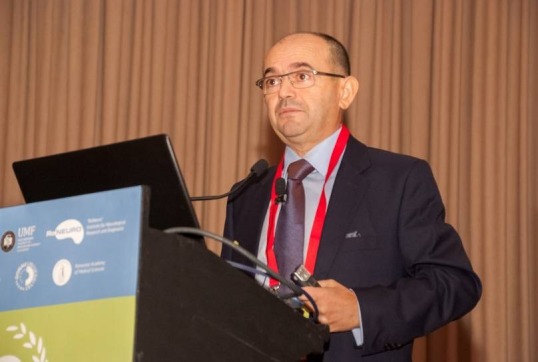
President of SSNN and the Neurology Society in Romania, Prof. Dafin Muresanu, MD

Moreover, the events that took place during the Congress have definitely confirmed its value. The very high number of specialists of international value, of over 200, from 15 countries (USA, Israel, Germany, Austria, Sweden, Italy, Russia, China, Egypt, Chile, Korea, Vietnam, Ukraine, Uzbekistan and Romania) have made it possible that this prestigious reunion of the medical elites in the field of medicine to be memorable, the presentations and the novelty of the approached subjects, raising the level of the Congress to excellence standards. The overwhelming personality and the huge experience of the moderators have also contributed to the success of the event.

**Fig. 2:**
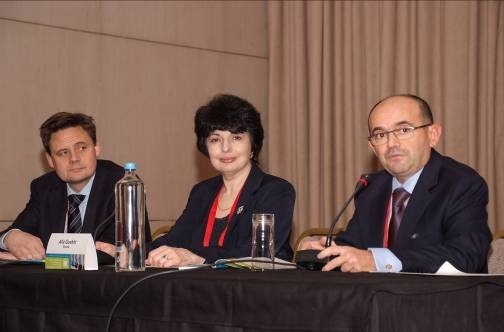
Discussions: Matthias Endres (Germany),Alla Guekht (Russia), Dafin Muresanu (Romania) - from left to right

**Fig. 3:**
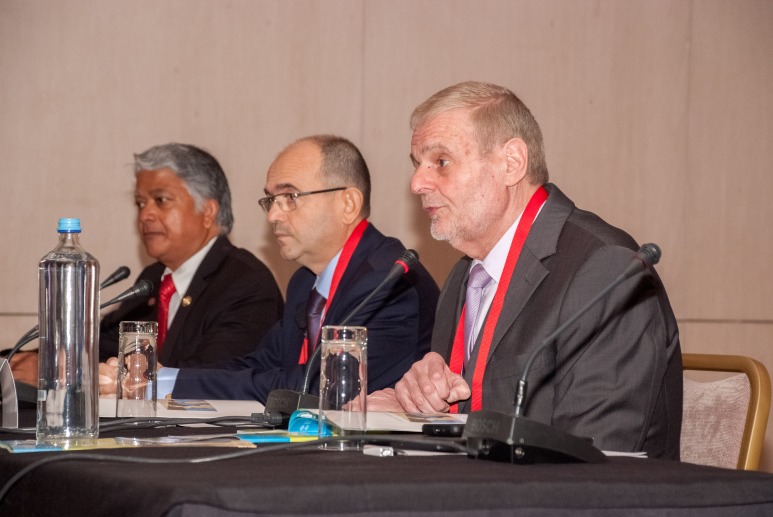
Welcome Address: Hari Shanker Sharm (Sweden), Dafin Muresanu (Romania), Natan Bornstein (Israel) - from left to right

Also, the exceptional professional value of the presentations had an important contribution to the Congress.

**Fig. 4:**
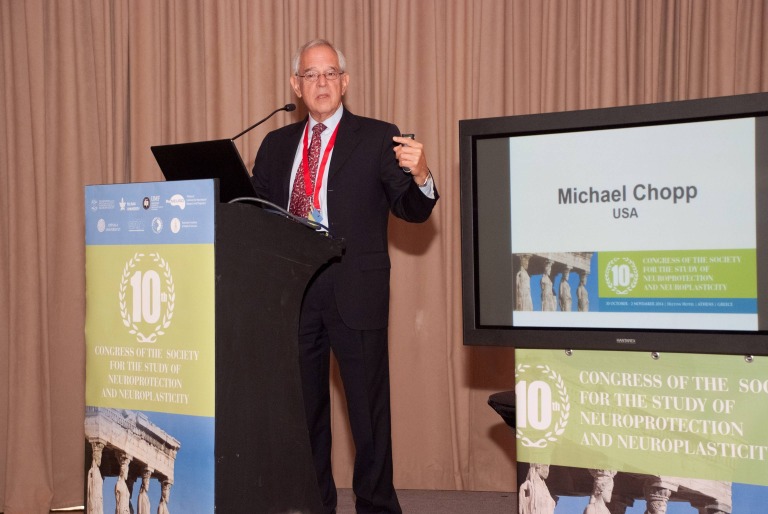
Michael Chopp (USA) Presentation: Neurorestorative treatments of experimental TBI

**Fig. 5:**
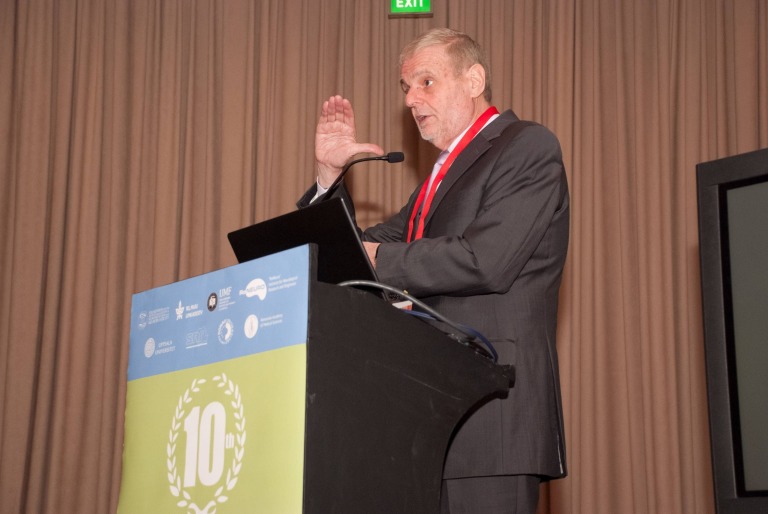
Natan Bornstein (Israel) Presentation: Early mobilization following stroke

**Fig. 6:**
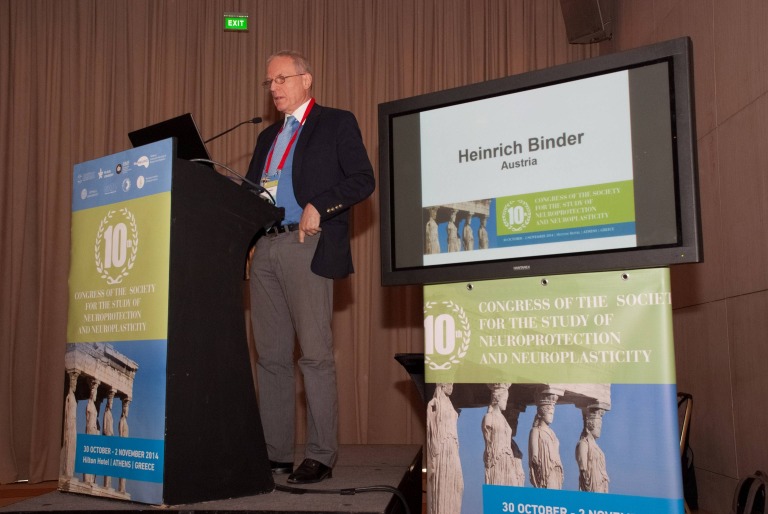
Heinrich Binder (Austria) Presentation: How neurorehabilitation benefits from cooperation among visionscience and neuroscience?

**Fig. 7:**
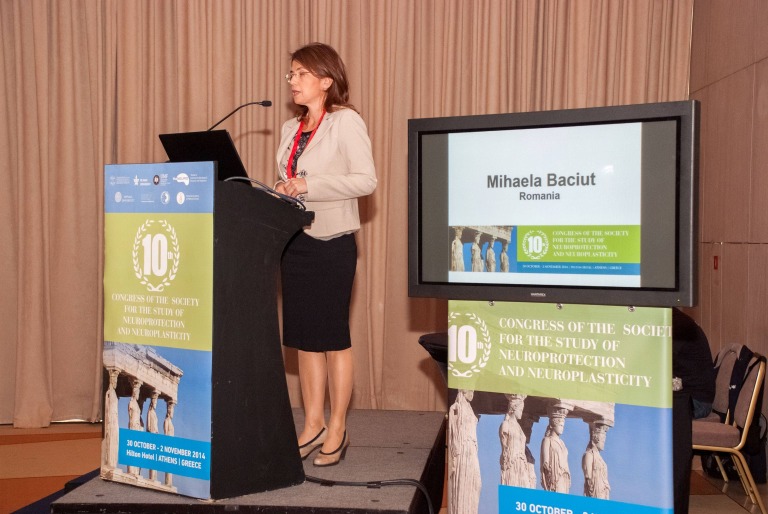
Mihaela Baciut (Romania) Presentation: Can we control surgical nerve injury in maxillofacial surgery?

Subjects such as the following have raised the interest of all the participants, the discussions on the interesting subjects also continuing during the coffee breaks and lunch: The levels of endogenous neuromodulation in normal and pathological brain conditions; Early mobilization following stroke; Dose and time related neuroprotective effects of neurotrophic factors in concussive head injury; Neurorestorative treatments of experimental TBI; How neurorehabilitation benefits from cooperation among visionscience and neuroscience?; Can we control surgical nerve injury in maxillofacial surgery?; Beyond revascularization – comprehensive treatment of acute stroke; CASTA revisited – new insights into acute stroke treatment with neurotrophic factors; A clinical-genetic algorithm for calculating the stable therapeutic dose of acenocoumarol; CADASIL and CARASIL: update on the clinical and molecular aspects; Of mice and man: Modelling post-stroke depression experimentally; Enhancing restoration after stroke: therapeutic approaches; Towards a multidimensional approach in clinical neuroscience research – advances and challenges; Mast cells and amyothropic lateral sclerosis: at the crossroads?; The modern treatment of Parkinson’s disease; Lack of innovation in neuropsychopharmacotherapy, The non-motor Parkinson’s disease; clinical and therapeutic approach; CLOCK genes and neuropsychiatric disorders.

Benefiting from an excellent organization and an impeccable organizational team, the Congress ended in a symbiosis note between science and culture, which has become a tradition. This way, the participants could enjoy visiting some of the most important locations in this legendary area, from the historical and cultural point of view, such as: the Olympic Stadium, Presidential Palace, Acropolis (ancient temples – Propylea, Parthenon and Erechteion), Acropolis Museum, The Arch of Hadrian, The Temple of Olympian Zeus, The House of Parliament, The Academy of Athens, The University, The National Library, The Omonia Square and The National Museum of Archaeology.

**Executive Editor** **Assoc. Prof. Dr. Eng. Victor Purcarea**

